# Assessing food and nutrition literacy in children and adolescents: a systematic review of existing tools

**DOI:** 10.1017/S1368980021004389

**Published:** 2022-04

**Authors:** Nicholas Carroll, Maude Perreault, David WL Ma, Jess Haines

**Affiliations:** 1 Department of Human Health and Nutritional Sciences, University of Guelph, Guelph, ON, Canada; 2 Department of Family Relations and Applied Nutrition, University of Guelph, 50 Stone Road East, Guelph, ON N1G 2W1, Canada

**Keywords:** Food literacy, Nutrition literacy, Children, Adolescents, Systematic review

## Abstract

**Objective:**

Food literacy (FL) and nutrition literacy (NL) are concepts that can help individuals to navigate the current food environment. Building these skills and knowledge at a young age is important for skill retention, confidence in food practices and supporting lifelong healthy eating habits. The objectives of this systematic review were to: (i) identify existing tools that measure FL and NL among children and/or adolescents and (ii) describe the psychometric properties.

**Design:**

A 4-phase protocol was used to systematically retrieve articles. The search was performed in May 2021. Study characteristics and psychometric properties were extracted, and a narrative synthesis was used to summarise findings. Risk of bias was assessed using the COSMIN checklist.

**Setting:**

Six databases were searched to identify current tools.

**Participants:**

Children (2–12 years) and adolescents (13–18 years) participated in this study.

**Results:**

Twelve tools were identified. Three tools measured FL, 1 tool measured NL, 4 tools measured both FL and NL, and 4 tools measured subareas of NL—more specifically, critical NL, food label and menu board literacy. Most tools were self-reported, developed based on a theoretical framework and assessed some components of validity and/or reliability for a specific age and ethnic group. The majority of tools targeted older children and adolescents (9–18 years of age), and one tool targeted preschoolers (3–6 years of age).

**Conclusions:**

Most widely used definitions of FL and NL do not acknowledge life-stage specific criterion. Continued efforts are needed to develop a comprehensive definition and framework of FL and NL appropriate for children, which will help inform future assessment tools.

There has been a global shift in food systems—whereas highly processed, low cost and convenient food items are more widely available in comparison to foods that are minimally processed and nutrient rich^(1)^. The availability of these highly processed foods, which are typically nutrient poor and energy dense, make it challenging to navigate the current food landscape to access and select nutritious foods.

Food literacy (FL) and nutrition literacy (NL) are concepts that may be important factors in supporting healthful dietary habits. While the literature includes a variety of definitions for FL and NL and their corresponding constructs^(2–4)^, NL has been defined as ‘*the degree to which individuals have the capacity to obtain, process, and understand nutrition information and skills needed in order to make appropriate nutrition decisions*’^(5)^; where FL has been defined as ‘*the scaffolding that empowers individuals, households, communities or nations to protect diet quality through change and strengthen dietary resilience over time. It is composed of a collection of inter-related knowledge, skills and behaviours required to plan, manage, select, prepare and eat food to meet needs and determine intake*’^(6)^. In this sense, being more food and nutrition literate provides one with the necessary aptitude and abilities to help navigate our current food environment. Indeed, FL and NL have been identified as key components in the promotion and maintenance of healthy dietary practices^(7–9)^. Both FL and NL are included in this review to provide a comprehensive assessment of existing tools^(8)^.

Supporting children in developing FL and NL, starting as early as in preschool years, can shape their food choices—since building these aptitudes and capabilities at a young age may be important for skill retention^(10)^, confidence in food practices^(11)^ and supporting healthy eating habits later in life^(12,13)^. A 10-year longitudinal study observed that those with higher cooking skills in emerging adulthood prepared meals with vegetables more frequently and consumed fast food less often as adults^(12)^. Research has also shown that, among a sample University students, the strongest predictor of food skills was meal preparation as a teenager^(14)^. While these studies underscore the importance of acquiring FL and NL early in life, there is currently no universally accepted tool to measure either FL or NL among children and adolescents.

NL and FL have been studied more extensively in the adult population, and a systematic review by Yuen, Thompson & Gardiner^(15)^ identified 13 tools targeting adults. In addition, a scoping review by Amouzandeh and colleagues^(16)^ summarised the psychometric properties of 12 instruments that were used to assess FL among adults. While these reviews identified existing measures of FL and NL in adults, to our knowledge, no systematic review has been conducted to identify tools for children and adolescents. To ensure they are appropriate for use, NL and FL assessment tools must undergo validity testing, that is, to assess the extent to which the measure accurately reflects the intended variable, in addition to reliability testing, which refers to the consistency of a measure. Identifying valid and reliable tools that assess FL and NL in childhood can help facilitate research that examines the long-term impact of building these skills, abilities and aptitudes early in life.

The objectives of this systematic review were to: (i) identify existing tools that measure FL and NL among children and/or adolescents and (ii) describe the validity and reliability of these tools. Findings from this review will help to identify tools for use in FL and NL research with children as well as identify potential gaps in existing FL and NL measurement.

## Methods

This systematic review was conducted in accordance with the PRISMA (Preferred Reporting Items for Systematic Reviews and Meta-Analyses) guidelines^(17)^. This systemic review was registered with PROSPERO (CRD42021241819).

### Search strategy

A systematic search of the literature was completed by 2 review authors (N.C. and M.P.). The search was last performed in May 2021. The following 6 databases were used: PubMed, Ovid MEDLINE, ScienceDirect, Web of Science, CINAHL plus and PsycInfo. Key words were identified through the expert guidance of a research librarian in addition to being based on previous literature reviews^(15,16,18)^. The search terms included: preschool* OR child* OR adolescen* OR teen* AND ‘food literacy’ OR ‘food skills’ OR ‘nutrition literacy’. The complete search strategy for each database is provided on the Online Supplementary Table S1. Forward citation searching was also used on the articles included in the final review.

### Eligibility criteria

Articles were included if they were primary research, described the development of a tool explicitly assessing FL or NL and assessed some form of validity (including content validity, face validity or structural/construct validity) or reliability (including internal consistency or test–retest reliability) testing. Only tools targeting children (2–12 years old) and/or adolescents (13–18 years old) were included. In the case that the ages extended beyond these ranges, if the mean age of the sample was ≥2 or ≤18 years, articles were included. Publications were excluded if an English language version was not available and if they were grey literature (e.g. theses, reports).

### Study selection

A 4-phase screening process was applied to identify articles to be included in the review. After completing the search, articles were exported and saved into Mendeley (Version 1.19.4). Duplicates were removed. The titles and abstracts of the remaining articles were then screened by 2 reviewers (N.C. and M.P.), independently. After excluding articles based on the eligibility criteria in review of the titles and abstracts, the remaining studies were then further examined by a review of the full text. Any differences across reviewers (N.C. and M.P.) throughout the screening and selection process were discussed and resolved.

### Data extraction and analysis

From each article that was included in the review, the following information was extracted into a table: (1) key characteristics of the identified tools (author, year of publication, country of origin, tool name, purpose, conceptual framework, number of items and constructs assessed, scoring details, method of development, method of administration, sample characteristics); and (2) psychometric properties (content validity, face validity, structural/construct validity, reliability). Missing or unclear information was also identified and reported. One review author (N.C.) extracted the data and another author (M.P.) reviewed the data. A narrative synthesis of the included studies was conducted to summarise and analyse findings.

### Risk of bias assessment

Risk of bias was assessed using the COSMIN risk of bias checklist^(19)^. The assessment was completed independently by 2 reviewers (N.C. and M.P). Answers were compared and discussed to reach consensus. The COSMIN checklist consists of ten areas of consideration: outcome measure development, content validity, structural/construct validity, internal validity, cross-cultural validity, reliability, measurement error, criterion validity, hypothesis testing for construct validity and responsiveness^(19)^. Each area includes different items that are assessed using the following scoring: ‘Very Good’, ‘Adequate’, ‘Doubtful’, ‘Inadequate’ or ‘Not Applicable’^(19)^. The score ‘Not Reported’ was indicated when the details could not be found in the article. For each area of consideration, the lowest score across the area items was used to evaluate the overall quality of the measurement property^(19)^. Because the COSMIN checklist was developed for evaluating tools designed to measure symptom or functional health status, the detail and rigour described for the outcome measure development and content validity areas of the checklist exceeded what is typically presented for assessment of health behaviours, such as FL and NL. Thus, these areas of the checklist were not assessed, and were instead narratively summarized.

## Results

**Fig. 1:**
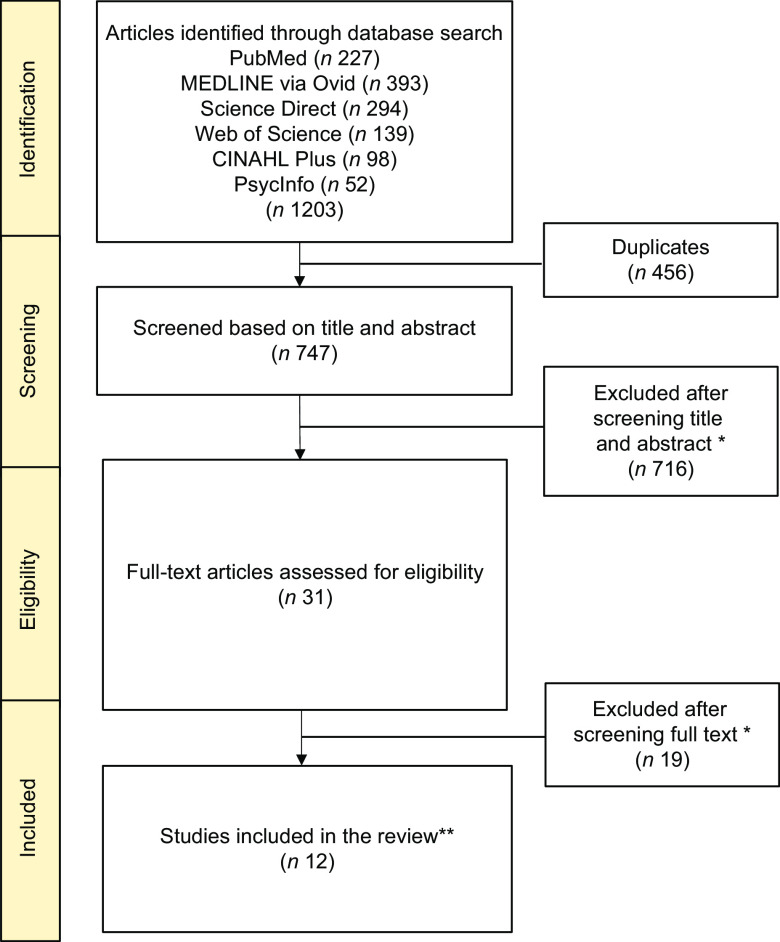
Flowchart of the literature search and review process *The reasons for exclusion included: the article was outside the scope of the review (*n* 445), not a primary research article (*n* 128), did not include children or adolescents as the study population (*n* 102), did not provide information on the development or validation of the food literacy (FL) or nutrition literacy (NL) measure (*n* 45), or not in English (*n* 15). **One tool was identified in the forward citation step.

After the 4-phase protocol, a total of 12 tools were included in this systematic review (Fig. 1). In the initial search, 1203 articles were identified, of which 747 remained once duplicates were removed. Upon screening the titles and abstracts, 716 were excluded and the remaining 31 were assessed for eligibility by reviewing the full text. After the full-text review, 19 articles were excluded. The reasons for exclusion included: the article was outside the scope of the review (*n* 445), not a primary research article (*n* 128), did not include children or adolescents as the study population (*n* 102), did not provide information on the development or validation of the FL or NL measure (*n* 45), or not in English (*n* 15). One article was retrieved in the forward citation step (*n* 1). Some studies appeared to meet the inclusion criteria, but were excluded, such as the study by Gartaula *et al*.^(20)^. While FL was evaluated among students, their definition only considered knowledge relating to cultivation practices and the processing of finger millet^(20)^. In addition, multiple publications^(21–24)^ have been released using the Preschool-FLAT and the FNLIT; where, only the validation studies^(25,26)^ were included in this review.

### Summary of existing tools

The 12 tools that assessed FL and NL among children and adolescents are summarized in Table 1. Three of the 12 tools measured FL^(26–28)^, 4 tools measured both FL and NL^(25,29–31)^, and the other 5 tools measured NL or subareas of NL^(32–35)^. More specifically, 2 of the NL tools assessed critical NL^(34,35)^, 1 of the tools measured menu board literacy^(33)^ and the other tool assessed food label literacy^(36)^. Critical NL focuses specifically on critically appraising and understanding nutrition information^(34)^, while menu board literacy targets understanding of menu board nutrition information^(33)^ and food label literacy measures one’s ability to interpret the information on food labels^(36)^. The studies describing the 12 tools were published between 2012 and 2021 and were developed in 7 geographical regions: 2 from Norway^(34,35)^, 3 from the USA^(28,33)^, 1 from Denmark^(27)^, 1 from Thailand^(32)^, 3 from Iran^(25,30,31)^, 1 from Italy^(26)^ and 1 from China^(29)^. The purpose of most of the studies was to develop and test the validity and/or reliability of a tool that assesses either FL or NL. However, 2 of the studies^(27,35)^ tested the validity and reliability of a previously developed tool, while one study^(30)^ modified and updated a previously existing tool^(25)^. The tools were generally tailored to a specific population. For instance, the TFLAC^(28)^ was developed for low-to-middle income, ethnically and racially diverse school-aged children living in the USA (grades 4–5). One tool was intended for preschoolers (3–6 years of age)^(26)^, 5 tools were designed for school-aged children (7–12 years of age)^(25,28,30,33,36)^, and 5 tools were aimed at adolescents (12–18 years of age)^(27,31,32,34,35)^. One tool was developed for school-aged children and adolescents (7–17 years of age) but was only validated among adolescents between ages 13–15 years^(29)^.

**Table 1:** Summary characteristics of the food literacy and nutrition literacy tools for children and adolescents

Author (year), country of origin	FL or NL	Tool name	Purpose	Conceptual framework	Number of items and constructs assessed	Scoring details	Method of development	Method of administration	Sample characteristics
Khorramrouz (2021), Iran	FL and NL	Modified Food and Nutrition Literacy (M-FNLIT)	To update the previous version of the FNLIT questionnaire in upper primary schoolchildren in Mashad	Not specified, assumed to be based on the same conceptual framework as the FNLIT tool (Nutbeam’s model^(37)^)	40 items measured under 2 domains with 6 subscales: cognitive (understanding food and nutrition information, nutrition health knowledge); skills (functional, interactive, food choice literacy, critical)	36 Likert-type items and 4 true-false questions. Scores of ‘1’ to ‘5’ were allocated to the response of items, except items 9–15 were inversely scored. True-false questions were dichotomized, whereas a score of ‘1’ for incorrect and ‘5’ for correct responses. Total raw scores range from 40 to 200 which were then proportionally transformed to a total between 0 and 100	A 4-phase process was applied: Phase 1: content and face validity of the questionnaire using Delphi consensus and interviewing; Phase 2: construct validity assessed; Phase 3: Internal consistency and reliability evaluated; Phase 4: Detect cut-off scores of the M-FNLIT scale	Self-reported survey in paper and pen format	Sampled included (*n =* 319) students aged 9–12 years of age where 48·9 % were girls. Majority of sample had a normal BMI z-score (boys: 59·5 %, girls: 56·4 %) where 20·9 % of boys were obese and 25 % of girls were overweight. In addition, most of the sample’s mother and father were mid-to-highly educated, only a small proportion were either illiterate or had less than or equal to 5 years of education. 64 students took part in the test–retest assessment
Liu (2021), China	FL and NL	Food and Nutrition Literacy Questionnaire for Chinese School-age Children (FNLQ-SC)	To develop and validate a questionnaire to assess the food and nutrition capacity of children and provide targets for further nutrition education and intervention	Nutbeam’s model^(37)^: functional nutrition literacy, interactive nutrition literacy, critical nutrition literacy	50 items measured under 2 domains with 5 subscales: knowledge and understanding (knowledge and understanding of food and nutrition); skill (access to and planning for food, selecting food, preparing, eating) with 19 components	Questions included 5-point Likert type questions (e.g. “I am concerned about nutrition and health information: never, seldom, sometimes, usually, always”), choice questions (e.g. ‘Which of the following snacks is healthier?’), and fill-in-the-blank questions (e.g. ‘Fill in your height and weight.’). Each question was scored based on 2 points. Highest possible score for students of grade 7–8 is 100, while grades 5–6 is 98 (1 question skipped) and grade 3–4 is 92 (4 question skipped)	A 2-phase process was applied: Phase 1: Construction of food and nutrition literacy core components for school-aged children (including literature review and expert interview using Delphi consensus); Phase 2: Questionnaire development (evaluating appropriateness, readability and difficulty)	Self-reported, did not detail method of administration	Sample included students aged 7–17 years (*n* 2452) for the reliability and validity study. Most of the students were 13–15 years of age (92·6 %), and about half were female (49·6 %). Family affluence status with 13·6 % as poor, 53·3 % as medium and 32·1 % as affluent. Caregiver’s education level was mostly (49·1 %) junior high school and roughly 52·8 % have received nutrition education at school
Stjernqvist (2021), Denmark	FL	Food Literacy Instrument	To develop, test, and validate an instrument to measure food literacy in schoolchildren aged 11–15 years	Deductive approached based on Benn’s 2014^(38)^	37 items and 5 constructs: to know (understanding of coherence); to do (everyday life competencies, practical and technical); to sense (sensory competencies in cooking and tasting); to care (ethical considerations); and to want (citizenship, responsibility and willingness)	35 Likert-type items and 2 true/false items. Each competency has its own response options (e.g. to sense: have not tried this – very difficult – difficult – easy – very easy; or to know: correct – wrong – do not know). No information on scoring details; but higher scores indicates more food literate	A 3-phase process was applied with 8 steps: Phase 1: development by experts (content validity); Phase 2: scale testing (face validity, sampling and survey administration, item analysis); Phase 3: validation (test of dimensionality, reliability, and validity)	Self-reported questionnaires administered electronically during a single school lesson	Sample included (*n* 817) students in grades 6–7 where 55 % were girls. Little over two-thirds of the sample were from public schools while the latter were from a private school. About half of the sample took part in the development (*n* 409), and the validation (*n* 408) while a smaller subset (*n* 267) took part in retest
Ashoori (2020), Iran	FL and NL	Food and Nutrition Literacy Assessment Tool (FNLAT)	To develop and validate a FNLAT for high-school graduates and young adults in Iran	Nutbeam’s model^(37)^: functional nutrition literacy, interactive nutrition literacy, critical nutrition literacy	60 items measured under 2 domains with 6 subscales: knowledge (food and nutrition knowledge); skills (functional, interactive, advocacy, critical analysis of the information, food label and reading skills)	Questions included binary questions (e.g. assessing food and nutrition knowledge and food label reading skills) and Likert type questions (e.g. assessing skill domain)	A 5-phase process was employed: Phase 1: Identification of FNL components for high school graduates and youth; Phase 2: Item generation and drafting the questionnaire; Phase 3 Assessment of content and face validity; Phase 4 Assessment of construct validity; and Phase 5 Assessment of reliability of the developed questionnaire	Self-reported, did not detail method of administration	Sampled included (*n =* 697) students aged 17–18 years of age where 51·5 % were female. The majority (52·2 %) were from high SES city districts, while others were from middle SES (24·7 %) and low SES (23·1 %) city districts. 28 students took part in the test–retest assessment
Deesamer (2020), Thailand	NL	Thai–Nutritional Literacy Assessment Tool for Adolescents (Thai–NLAT)	To develop the Thai–NLAT and test its validity and reliability	Nutbeam’s model and Velardo’s concept^(37)^: functional nutrition literacy, interactive nutrition literacy, critical nutrition literacy	61 items assessing 5 constructs: macronutrients–micronutrients and health; nutrition and energy balance; decision–making on nutrition information; food processing; food safety	Scored out of a maximum of 61 points: 3 choices scale; incorrect responses scored as ‘0’ and correct responses scored as ‘1’ with scores summed with a higher final score indicating better nutrition literacy	A 2-phase process was employed: Phase 1 (Steps 1–5) qualitative interviews with experts to generate tool items, and preliminary item tryout (content validity, face validity, internal consistency); Phase 2 (Step 6): psychometric tests (concurrent validity, construct validity)	Self-reported, did not detail method of administration	Step 5: preliminary item tryout from (*n =* 275) Thai adolescents in grade 7–9 and grade 10–12 in public schools Bangkok Step 6: psychometric testing among (*n* 442) Thai adolescents with a mean age of 14·7 ± 1·8 years and 57·9 % were males. Majority were normal weight (76·9 %) and some were obese (13·3 %), overweight (6·1 %) and underweight (3·6 %)
Tabbachi (2020), Italy	FL	Preschool–Food Literacy Assessment Tool (Preschool–FLAT)	To assess the validity and internal consistency of the Preschool–FLAT	Vidgen’s description of FL knowledge and skills components^(6)^	20 items assessing 5 constructs: relationship between weight status and food/health; relationship between food quality/quantity and health, and knowing the main food categories; relationship between food and environment; traditional foods; distribution of foods at different daily meals	Scored out of a maximum of 20 points; each domain measured on a 5-point Likert scale (0–4): ‘0’ indicates no food literacy and ‘20’ indicates high food literacy	The Preschool–FLAT was previously developed by the Training-to-Health team. The following measures were assessed in this study: content validity (expert panel); internal consistency (Cronbach’s *α*); construct validity (structural equation modeling); discriminant validity (intervention *v*. control group)	Assessed by the educator in paper and pen format	Sample included (*n* 505) preschoolers. 53·7 % were male, most were 5–6 years (44·4 %), 4 years (39·6 %) and the remaining were 3 years (16·0 %). Half of the sample were from a low/medium school SEE (55·8 %) and medium/high school SEE (44·2 %). A majority of the children were normal weight (66·5 %) with a mean BMI of 16·3 kg/m^2^(sd 2·5)
Amin (2019), US	FL	Tool for Food Literacy Assessment in Children (TFLAC)	To develop and describe the content validity and reliability of a food literacy assessment tool among low-to-middle income, ethnically and racially diverse school-aged children (grades 4–5)	Not specified	25 items assessing 5 constructs: cooking skills; cooking knowledge; nutrition knowledge; food systems knowledge; and self-efficacy regarding eating	Scored out of a maximum of 40 points (details not provided): higher a score indicates more food literacy while a lower score represents less food literacy	A 3-phase process was used: Phase 1: content validity (Delphi panel with experts, content validity ratios); Phase 2: Tool Pilot study among children (feedback on each question); Phase 3: internal consistency (Cronbach’s *α*) and test-retest reliability (intraclass correlation coefficient) among children	Self-reported survey in paper and pen format	Phase 2: children from 2 Massachusetts elementary schools (*n* 38; grades 4–5) both sets were non-white (45 % and 66 %) and lower socioeconomic status (40 % and 52 %); Phase 3: children (*n* 706; aged 9–11) from 12 low-to-middle SES (26 %–65 %) racially and ethnically diverse elementary schools and afterschool programs
Naigaga (2018), Norway	NL	Critical Nutrition Literacy Scale (CNL–E)	To examine the psychometric properties of the CNL-E scale to measure adolescents’ perceived proficiency in ‘critically evaluation nutrition information from various sources’	Nutbeam’s model^(37)^: functional nutrition literacy, interactive nutrition literacy, critical nutrition literacy	5 items and 2 constructs: the extent of trusting nutrition information from different sources (items 1–3); and the proficiency to establish the falsifiability of nutrition claims by judging the information against basic knowledge nutrition (items 4–5)	Scored out of a maximum of 30 points with responses scaled on a 6-point scale from ‘very difficult’ as ‘1’ to ‘very easy’ as ‘6’: higher scores represent higher perceived proficiency in critically evaluating nutrition information	The Rasch analysis approach was used to examine the psychometric properties of the CNL-E; as well as multidimensional Rasch modelling and confirmatory factor analysis	Self-reported questionnaire using an electronic survey system	Sample included (*n* 1622) students aged 15–16 years in grade 10 from roughly 60 schools in Norway. Students reported: gender, predominant language, place of birth, and an indicator of socioeconomic status; however, the article did not detail such information
Doustmohammadian (2017), Iran	FL and NL	Food and Nutrition Literacy (FNLIT)	To develop and test the validity and reliability of a questionnaire that assess food and nutrition literacy in elementary school children in the city of Tehran	Nutbeam’s model^(37)^: functional nutrition literacy, interactive nutrition literacy, critical nutrition literacy	46-items measured under 2 domains with 6 subscales: cognitive (knowledge and understanding); skills (functional, food choice, interactive, and critical skills)	42 Likert-type scale and 4 true/false items. Did not mention the scoring details	A 3-phase process was applied: Phase 1: literature review and qualitative study to identify food and nutrition literacy dimensions and scale items; Phase 2: development and validation of the scale (item generation, content validity, face validity, construct validity, reliability); Phase 3: Confirmatory study	Self-reported, did not detail method of administration	Phase 2: construct validity study among (*n* 373) students in grade 5 (48 %) and 6 (52 %) with a mean age of 11·1 ± 0·6 years and about half were male (51 %). Phase 3: confirmatory study among (*n* 400) students aged 10–12 years (11·3 ± 0·6) and similarly, roughly half were male (51 %). Both studies also described their sample in terms of educational districts based on three socioeconomic levels
Williams (2017), US	NL	Menu Board Literacy (MBL) Instrument	To develop and measure the psychometric properties of an instrument that assesses menu board literacy in children	Not reported	27 items and 2 constructs: menu board literacy (20 items) and self-efficacy (7 items)	It is presumed that the menu board literacy items had one correct answer while the self-efficacy items were assessed on a 5-point Likert scale (‘Definitely cannot do this’ to ‘Extremely Confident’). However, the study did not provide details on the scoring	A 2-phase process was employed: instrument development (review, generation of items, content validity by panel of experts, cognitive interviews to students to generate final set of items) and assessment of reliability/readability (internal consistency both pretest and post-test, the Flesch Reading Ease Index)	Self-reported measure in paper and pen format; students were not able to use a calculator	Phase 1: cognitive interviews on (*n* 24) Black and Hispanic 4th and 5th graders Phase 2: 2 convenience samples of similarly representative students (*n* 32 and 141, respectively). Children were recruited from low-income New York City neighborhoods
Guttersrud (2015), Norway	NL	Engagement in Dietary Behaviour (EDB) scale and Self-Efficacy (se) in Science Scale	To assess the appropriateness of using latent scales to measure critical nutrition literacy	Nutbeam’s definition of Nutrition Literacy^(37)^ Schwarzer and Fuchs^(39)^ social-cognitive model of health behavior change, including self-efficacy	8-items in the engagement in dietary behaviour scale (Engagement Scale); 11-items in the critical stance towards nutrition claims (Claims Scale)	Six-point rating scale (strongly disagree, to strongly agree) for both scales	The Rasch model was used to assess item discrimination, model fit, reliability and targeting – with the purpose in constructing a valid and reliable measure	Self-reported measure using and electronic survey system	Sample included (*n* 740) tenth-grade students with an age range from 14–15 years where 48 % of the students were females and 9 % were minorities
Reynolds (2012), United States	NL	Food Label Literacy for Applied Nutrition Knowledge (FFLANK)	To determine the reliability and validity of a 10-item questionnaire, the FLLANK	Not specified	10-items assessing the ability to make healthful food choices based on the Nutrition Facts and ingredients lists found on food labels	Scored by the percentage of correctly identified products with response options including: ‘Label A’, ‘Label B’ or ‘Don’t Know’	The FLLANK was designed by the developers of nutrition knowledge intervention, titled Nutrition Detectives. The tool was administered 4 times: before and after the program, and then 3 months later, before and after the additional session	Self-reported measure in paper and pen format; teachers are asked to evaluate the answers with the answer sheet provided	Sample included (*n* 499) elementary school aged children (mean age 8·6 ± 0·9 years) from grades 2–4 where 51 % of the students were girls

FL, food literacy; NL, nutrition literacy.

While 3 of the studies did not specify a conceptual framework or model on which the measure was based^(28,33,36)^, 7 of the tools assessing NL^(25,29–32,34,35)^ were based on Nutbeam’s health literacy model^(37)^ and 2 were based on the models of FL^(26,27)^ by Benn^(38)^ and Vidgen and Gallegos^(6)^, respectively. Among the 5 tools that focused on NL, the number of constructs assessed in the tools ranged from 2 to 5 and the number of items ranged from 5 to 61. The Thai–NLAT^(32)^ was the most comprehensive in assessing NL with 5 constructs: macronutrients–micronutrients and health, nutrition and energy balance, decision–making on nutrition information, food processing and food safety. The number of constructs assessed in the FL tools and the 4 FL and NL tools ranged from 5 to 6 and included from 20 to 60 items. Most of the tools included similar constructs including nutrition-related knowledge as well as functional skills, like cooking skills. An exception was the Preschool–FLAT^(26)^, which assessed concepts that are not covered in other measures, such as assessing the relationship of body weight and food and children’s ability to assemble traditional foods to create a meal.

Four tools^(25,29,31,32)^ did not report the method of administration, 5 tools^(26,28,30,33,36)^ used a paper and pen format, while 3 tools^(27,34,35)^ were administered electronically. All tools were self-reported by the students except for the Preschool–FLAT^(26)^ which was interview led by the child’s educator.

Scoring differed across tools including a combination of either Likert-scaled or true or false, and correct or incorrect questions. Five out of 11 tools incorporated task-based items, e.g. creating a healthy meal using images^(26,27,29,33,36)^. Some of the tools did not indicate how the score was interpreted^(25,31,33,35)^ but for the most part, higher scores were indicative of higher FL or NL.

### Validity and reliability of existing tools

Nine^(25–33)^ out of the 12 identified tools underwent content validity testing by a panel of experts including 5–29 experts (Table 2). Three tools^(28–31)^ specifically assessed content validity using a modified Delphi approach with experts. Six^(25,27,30–33)^ out of the 12 tools assessed face validity, by either conducting interviews^(25,30–33)^ or focus groups^(27)^ with their intended target population of the tool. Ten out of the 12 tools assessed structural/construct validity either using the Rasch model^(34,35)^, confirmatory factory analysis^(25,27,29–31)^, the Known Group technique^(32)^, Structural Equation Modelling^(26)^ or between-group differences based on ONQI scores^(36)^. Some tools also assessed convergent validity using a health literacy instrument^(27)^, using food intake as an outcome^(27)^ or using a Healthy Eating Index score^(32)^. One tool was assessed for its ability to detect change following a FL intervention^(26)^, while 2 tools measured change after a specific nutrition knowledge program^(33,36)^.

**Table 2:** Validity and reliability of the reviewed food and nutrition literacy tools for children and adolescents

Tool author (year)	Validity			Reliability	
	Content validity: covers all constructs	Face validity: appears to measure construct of interest	Structural/construct validity: measures the concept intended to measure	Internal consistency: measures if several items measure the same construct	Test-retest reliability: measures the consistency of a test over time
M-FNLIT Khorramrouz (2021)	Content validity assessed and evaluated by 2-round Delphi consensus of 20 experts Findings from 2-rounds (respectively): CVR: 0·72 and 0·92 CVI: 0·92 and 0·98	Face validity assessed using a convenience sample of 10 children aged 9–12 years. Impact scores were also calculated by evaluating the frequency and importance of items	Structural/Construct validity assessed using confirmatory factory analysis to examine whether the data fits the measurement model	All 36 items: Cronbach *α* = 0·88 Internal consistency across 6 subscales ranged from *α* = 0·22–80	Test re-test all 36 items: ICC = 0·95 Test re-test of 6 subscales: ICC = 0·73–0·91
FNLQ-SC Liu (2021)	Content validity was assessed by the Pearson correlation coefficient Pearson correlation coefficients between each dimension and the overall questionnaire ranged from 0·370 to 0·877	Not reported	Structural/Construct validity assessed using exploratory factor analysis to explore whether the statements in the questionnaire reflected the conceptual framework, in addition to confirmatory factor analysis The Kaiser-Meyer-Olkin test showed sampling adequacy (KMO = 0·738), and Bartlett’s test confirmed that factor analysis was appropriate (*P* < 0·001)	All 50 items: Cronbach *α* = 0·70 Internal consistency across 5 subscales ranged from *α* = 0·15–0·45	Not reported
FL Tool Stjernqvist (2021)	Content validity assessed and validated by a group of experts based on Benn’s (2014) model of FL^(38)^	Face validity assessed using 2 focus group interviews with 12 schoolchildren	Structural/Construct validity assessed using confirmatory factory analysis in reducing items and comparing models Convergent validity assessed using a health literacy instrument for school-aged children (2016)^(40)^; significant positive association for the total FL scale (*β* = 9·82, *P* < 0·001) and its 5 competencies Convergent validity assessed using food intake as an outcome with a food frequency index (2014); significant association for the total FL scale (*β* = 2·32, *P* < 0·001) and its 5 competencies	All 37 items: Cronbach *α* = 0·85 Internal consistency across 5 subscales ranged from *α* = 0·50–0·73	Test re-test all 37 items: ICC = 0·92 Test re-test of 5 subscales: ICC = 0·76–0·88
FNLAT Ashoori (2020)	Content validity assessed by literature review, expert panels and evaluated by Delphi consensus of 19 experts CVI: >0·90	Face validity assessed using cognitive interviews among 10 high school students	Construct validity assessed using PCA and CFA. The Kaiser–Meyer–Olkin test showed sampling adequacy (KMO = 0·728), and Bartlett’s test confirmed that factor analysis was appropriate (*P* < 0·001).	All 60 items: Cronbach *α* = 0·84 Internal consistency across all subscales ranged from *α* = 0·71–0·82, except for the subscales ‘critical analysis of information’ (*α* = 0·64) and ‘food label reading skills’ *α* = 0·56)	Test re-test all 60 items: ICC = 0·93 Test re-test of 6 subscales: ICC = 0·59–0·90
THAI-NLAT Deesamer (2020)	Content validity assessed and validated by 7 experts based on Velardo’s (2015)^(9)^ concepts for NL in addition to assessing item-content validity	Face validity assessed using cognitive interviews among 10 Thai adolescents	Structural/Construct validity assessed using Known Group Technique; the healthy group (based on energy distribution and sugar intake) had nutrition literacy scores significantly higher than the unhealthy group Convergent validity assessed using the coefficient correlation analysis between the THAI-NLAT and the Thai Healthy Eating Index; significant positive relation between energy distribution and THAI-NLAT score of energy balance (*r* = 0·131, *P* = 0·021)	All 61 items: KR-20 = 0·83 Internal consistency assessed through item-subscale correlation, and ranged from *r* = 0·627–0·781(*P* < 0·01)	Not reported
Preschool-FLAT Tabacchi (2020)	Content validity assessed by a panel of 5 experts based on constructs adapted by Vidgen (2014)^(6)^ CVI = 0·94 CVR = 0·88	Not reported	Structural/Construct validity assessed using a SEM Discriminant validity assessed using a convenient intervention subgroup *v*. control group; unpaired *t*-test revealed statistical significance between FL score and those who received the intervention than those who did not receive it (mean 15·1 *v*. 7·1, *P* < 0·001)	All 16 items: Cronbach *α* = 0·77 Internal consistency across 4 subscales ranged from *α* = 0·73–0·76	Not reported
TFLAC Amin (2019)	Content validity assessed using 2-round modified Delphi approach with 16 panelists CVR Round 1 = 0·40 CVR Round 2 = 0·70	Not reported	Not reported	Internal consistency across all food literacy domains (except cooking knowledge) ranged from: Cronbach *α* = 0·80–0·98, with cooking knowledge: *α* = 0·63	Test re-test across all food literacy domains ranging from: ICC = 0·64–0·70 (*P* < 0·001)
CNL-E Naigaga (2018)	Not reported	Not reported	Structural/Construct validity assessed using the Rasch model showed a good fit with independent items, representing a well-targeted measurement	All 5 items: Cronbach *α* = 0·90	PSI = 0·88 for the original data set (with missing values)
FNLIT Doustmohammadian (2017)	Content validity assessed by a panel of 8 experts based on Nutbeam’s hierarchical model of health literacy^(41)^ CVI = 0·92 CVR = 0·87	Face validity assessed using interviews among 15 students	Structural/construct validity assessed using EFA and CFA. The EFA suggested a 6-factor construct with the CFA indicating acceptable fit indices among the proposed models	Internal consistency across the FNLIT scale and its subscales (except critical skill subscale) ranged from: Cronbach *α* = 0·71–0·80, with critical skill subscale *α* = 0·48	Test re-test all 46 items: ICC = 0·89 with its subscales ranged from: ICC = 0·78–0·91
MBL Tool Williams (2017)	Content validity assessed by a panel of 29 experts	Face validity assessed using cognitive interviews among 24 students	Not reported	Pilot sample: Reliability in all 20 MBL items (pretest): ω_t_ = 0·87, and (posttest) ω_t_ = 0·91, while 7 SE items (pretest): ω_t_ = 0·78, and (posttest) ω_t_ = 0·83 Internal consistency assessed by in MBL items: Ordinal *α* = 0·86 (pretest), and *α* = 0·90 (posttest) while SE items: Ordinal *α* = 0·78 (pretest), and *α* = 0·83 (postest)	Not reported
CNL Tool Guttersrud (2015)	Not reported	Not reported	Structural/Construct validity assessed using the Rasch model showed that the subscales measure the defined constructs	Internal consistency in the EDB scale and the SE in science scale were: Cronbach *α* = 0·86 and *α* = 0·92, respectively	The PSI were 0·79 and 0·90 for the EDB scale and the SE in science scale, respectively
FLLANK Reynolds (2012)	Not reported	Not reported	Structural/Construct validity assessed using between-group differences in ONQI scores generated from each product used in the FLLANK. Mean ONQI scores for the correct item responses were significantly higher than the mean ONQI scores that were incorrect (27·4 ± 9·4 *v*. 16·2 ± 9·4; *P* = 0·01)	All 10 items: Cronbach *α* = 0·77	Test re-test all 10 items: ICC = 0·68

CFA; confirmatory factor analyses; CVI, content validity index; CVR, content validity ratio; M-FNLIT, modified food and nutrition literacy; FNLQ-SC, food and nutrition literacy questionnaire for Chinese school-age children; FL, food literacy; THAI-NLAT, Thai-nutritional literacy assessment tool, preschool-FLAT, preschool-food literacy assessment tool; TFLAC, tool for food literacy assessment in children; CNL-E, Critical nutrition literacy-evaluation; MBL tool, menu board literacy tool; FLLANK, food label literacy for applied nutrition knowledge questionnaire; PCA, principal component analysis; SEM, structural equation model; EFA, explanatory factor analyses; ONQI, overall nutritional quality index; *α*, alpha; ICC, intraclass coefficient; KR-20, Kuder-Richardson-20; PSI, person separation index; ω_t_, McDonald’s omega; SE, self-efficacy; EDB, engagement in dietary behavior.

Seven tools reported overall internal consistency of their measure using Cronbach’s *α* ^(26,27,29–31,34,36)^ with internal consistency ranging from 0·77 to 0·90. Internal consistency across subscales was reported by 8 tools^(25–31,35)^, with internal consistency considered *acceptable to high* ^(42)^ for most tools’ subscales. The weakest internal consistency was observed in the subscale critical skill (*α* = 0·22) in the M-FNLIT tool^(25,30)^ and similarly, in the same critical skill subscale (*α* = 0·48) in the FNLIT tool^(25)^. The FNLQ-SC^(29)^ had lower than acceptable scores across all subscales (*α* = 0·15–0·45). One tool^(32)^ reported overall internal consistency using Kuder-Richardson 20 test, with internal consistency of KR-20 = 0·83, and one tool^(33)^ using ordinal *α* for their 2 subscales, ranging from 0·78 to 0·90.

Six tools^(25,27,28,30,31,36)^ reported test–retest reliability using Intraclass Correlation Coefficients (ICC) ranging from 0·64 to 0·93, suggesting *moderate to excellent reliability* ^(43)^. Two tools^(34,35)^ reported on test–retest reliability using Person Separation Index (PSI), with values ranging from 0·79 to 0·90, and one tool^(33)^ reported on McDonald’s omega on their 2 scales ranging from 0·78 to 0·91.

Ratings for the risk of bias assessment are presented in Supplemental Table S2. Most of the included studies showed a ‘Very Good’ methodological quality for the psychometric properties which were tested, including all 12 studies sufficiently evaluating internal consistency and 8 studies assessing structural/construct validity^(25–27,29–31,34,35)^. Moreover, 7 studies^(25,27,28,30,31,33,36)^ tested reliability; however, 3 articles^(27,28,33)^ had a narrow time window between test and retest (e.g. 1 week), where a ‘Doubtful’ score was subsequently given. Furthermore, Preschool–FLAT^(26)^ received an ‘Inadequate’ score for responsiveness, since there was insufficient information about the intervention. No studies reported cross-cultural validity, measurement error or criterion validity. Overall, all 12 studies generally showed low risk of bias.

## Discussion

This systematic review provides a comprehensive overview of published tools designed to measure FL and NL among children and adolescents. Twelve tools assessed some components of validity and/or reliability for specific target populations. The majority of tools targeted older children and adolescents (9–18 years of age), and one tool targeted preschoolers (3–6 years of age). FL and NL have primarily been studied among adults^(15,16)^ and research examining the use and applicability of these concepts among children and adolescents is still emerging. To our knowledge, this review is the first to systematically assess what FL and NL tools have been developed among children and adolescents.

All the tools underwent some testing of validity. Five tools were validated for most types of validity, including content, face and construct^(25,27,30–32)^, showing a rigorous process in designing and testing these tools. Most of the tools demonstrated acceptable to good internal consistency^(44)^. However, 6 tools^(25,27–29,31,32)^ had subscales with either poor or questionable internal consistency scores. This suggests that further adaptions may be needed to improve consistency among these subscales. Six out of 12 tools assessed test–rest reliability^(25,27,28,30,31,36)^ and were able to show that these tools could sufficiently replicate a similar result over time. Only 3 tools assessed whether they could detect change after an intervention or a nutrition education program^(26,33,36)^ and 2 of these tools focused specifically on sub-areas of NL. The Thai-NLAT^(32)^, the FNLAT^(31)^, the FNLIT^(25)^ and its adaptions^(30)^ have been shown to have the strongest psychometric properties. However, important factors need to be considered when using these tools, such as the context and culture within which these tools were developed and tested.

FL and NL are highly contextual^(3)^, influenced by the geography and its food system as well as the cultural and social context. The current tools have been developed for specific populations from countries around the world. For instance, the TFLAC^(28)^ has one item that focuses on food groups specific to their national food guidelines, whereas the FNLIT^(25)^ includes foods that are contextually and culturally appropriate such as Tafi, which is a type of chocolate. As suggested by Beaton *et al.* ^(45)^, a cross-cultural adaptation and validation of these existing tools is needed to ensure tools are appropriate for specific populations who might differ in language, cultural background as well as public health messaging.

In addition, FL and NL need to be conceptualised in an age-dependent manner, where younger children are not expected to develop the same level of complex skills as older teens or adults given their stages of cognitive development and reduced level of independence to make nutrition decisions. As mentioned, most of the existing tools targeted older children (9–18 years old), while only one tool targeted the preschoolers (3–6 years old). Tools developed for younger children such as the Preschool-FLAT^(26)^ are centered around general concepts like recognizing foods and building balanced plates. Using task-based approaches, specific questions that address these general concepts include: ‘Paint the foods suitable for lunch/dinner’, and ‘Compose a typical Sicilian meal by choosing foods among those shown’. On the other hand, tools developed for older teenagers such as the FNLAT^(31)^ focus on more complex nutrition knowledge and food choices that are more appropriate for this age group. Examples of questions include: ‘Which of the following foods are good sources of Calcium?’, and ‘If I go to grocery stores independently, I can easily ask the seller for the information I need.’ Given the small number of current tools available and the increase in FL and NL programs focusing on children and adolescents in recent years^(46)^, research should focus on developing and validating tools assessing FL and NL among children and/or adolescents, especially in younger children.

While FL and NL are related, there are important distinctions between their respective definitions. NL focuses largely on the knowledge and aptitude to obtain, interpret and use nutrition information whereas, FL is more comprehensive and encompasses a collection of inter-related knowledge, skills and behaviours, and extends to other determinants that may influence food decisions, like social and cultural factors^(4)^. This is reflected in the type of items and constructs included in the FL and NL tools. For example, 4 of the 12 tools included in this review measured both FL and NL^(25,29–31)^, their constructs assessed 2 universal domains, including both knowledge and skills. In comparison, the tools that only measured NL^(32–36)^, focused on cognitive competencies – for instance, Thai-NLAT^(32)^ included knowledge-based questions evaluating one’s understanding of nutrition information, food safety and energy balance.

Most definitions of FL and NL do not acknowledge life-stage specific criterion. The widely used definition of FL, conceptualised by Vidgen and Gallegos^(6)^, consists of 4 domains, some of which might not be applicable for children and adolescents. For instance, the plan and manage domain assesses one’s ability to make feasible food decisions in balancing personal needs, like nutrition, taste and hunger, with available resources^(6)^. This domain may not be appropriate for young children who have minimal independence over their food decisions. The Preschool–FLAT was informed by FL definition described by Vidgen and Gallegos^(6)^ and the authors stated they modified this definition to incorporate knowledge and skills suitable for preschoolers but provided limited detail on how those concepts were derived. While the emerging framework developed by Slater *et al.* ^(47)^ identified FL dimensions suitable for youth, none of the identified tools used this youth-focused framework. Continued efforts are needed to develop a comprehensive definition and framework of FL and NL appropriate for children. These efforts will help facilitate and inform developmentally appropriate assessment tools in the future.

An important limitation in the current tools assessing FL and NL among adults^(15,16)^ is the lack of objective, i.e. task-based, assessment of FL or NL. Five of the tools included in this current review used task-based assessment approach. The Preschool–FLAT^(26)^ incorporated visuals to assess certain FL constructs – for instance, one item asks the respondents to select which picture of food is the healthier option or which portion size is either small, medium or big. Similarly, the FLLANK assesses one’s ability to choose healthier food options given information from a nutrition facts label or an ingredients list^(36)^. The respondent is given 2 choices and is asked to determine which option is more nutritious. Incorporating task-based items can help reduce reporting and social desirability bias^(48)^ and, thus, future FL and NL measures for children should include both subjective, i.e. self or proxy reports and objective, i.e. task-based activities.

Findings from this review should be interpreted with some limitations. First, only articles that explicitly developed FL or NL assessment tools were included. It is possible that relevant articles may have been missed if they did not directly mention assessing literacy. However, our search strategy did include the term ‘food skills’ to help further yield an extensive pool of studies. Due to the variability and specificity across target audiences and the various frameworks used to inform their development, comparability across tools was limited. Despite these limitations, this review provides a current snapshot of existing tools assessing FL and NL among children and adolescents.

## Conclusion

Our systematic review identified 12 tools that measure FL or NL among children and adolescents in which 4 of these tools assess specific subareas of NL. The majority of tools targeted older children and adolescents (9–18 years of age) and only one tool targeted preschoolers (3–6 years of age). Most of the tools assessed validity or reliability within a specific target population. In addition, most FL and NL frameworks have been contextualised among adults. Considering cognitive development as well as constraints on children’s food decisions due to their limited independence, a comprehensive and developmentally appropriate definition and framework of FL and NL is needed for children. These efforts will help inform FL and NL assessment tools in the future^(9,39–41)^.

## Supplementary material

For supplementary material accompanying this paper visit https://doi.org/10.1017/S1368980021004389. click here to view supplementary material
