# Supplementary material for: Assessing food and nutrition literacy in children and adolescents: a systematic review of existing tools
Source: Public Health Nutr. 2021 Nov 3;25(4):850–65. doi: 10.1017/S1368980021004389 (PMC9991546; doi:10.1017/S1368980021004389)
Supplement: Supplementary file 1 [file S1368980021004389sup.zip › S1368980021004389sup002.docx]

| **Table S1** Search strategy for the systematic review | |
| --- | --- |
| **Database** | **Search Strategy** |
| *PubMed* | preschool* OR child* OR adolescen* OR teen* AND “food literacy” OR “food skills” OR “nutrition literacy” |
| *Ovid MEDLINE^1^* | preschool* OR child* OR adolescen* OR teen* AND "food literacy" OR "food skills" OR "nutrition literacy" |
| *ScienceDirect* ^2^ * | ((preschool OR child OR children OR adolescent OR adolescence OR teen) AND ("food literacy" OR “food skills” OR “nutrition literacy”)) |
| *Web of Science* * | ((preschool* OR child* OR adolescen* OR teen*) AND ("food literacy” OR “food skills” OR “nutrition literacy”)) |
| *CINAHL plus ** | ((preschool* OR child* OR adolescen* OR teen*) AND ("food literacy” OR “food skills” OR “nutrition literacy”)) |
| *PsycInfo ** | ((preschool* OR child* OR adolescen* OR teen*) AND ("food literacy” OR “food skills” OR “nutrition literacy”)) |
| ^1^ Combined with AND function  ^2^ Database does not support the use of wildcards ‘*’ and limited to a maximum of 8 Boolean connectors.  * Further specified source type to show results matching inclusion criteria (eg, include primary research articles and exclude grey literature). Databases specified as follows: ScienceDirect (show: Review articles and Research articles), Web of Science (show: Article, Review and Early Access), CINAHL plus (show: Academic Journals), and PsycInfo (show: Peer Reviewed Journal). | |

| **Table S2** COSMIN risk of bias checklist | | | | | | | | | | | | |
| --- | --- | --- | --- | --- | --- | --- | --- | --- | --- | --- | --- | --- |
| **Tool**  **Author (Year)** | **Concept** | | | | | | | | | | | |
|  | **Structural/Construct Validity** | **Internal Consis-tency** | **Cross-cultural Validity** | **Reliability** | **Measu-rement Error** | **Criterion Validity** | **Hypothesis Testing** | | **Responsiveness** | | | |
|  |  |  |  |  |  |  | **Conver-gent Validity** | **Discrimi-native or Known Groups Validity** | **Gold Standard** | **Other Outcome** | **Between Subgroup** | **Intervention** |
| **M-FNLIT**  Khorramrouz (2021) | V | V | NR | V | NR | NR | NR | NR | NR | NR | NR | NR |
| **FNLQ-SC**  Liu (2021) | V | V | NR | NR | NR | NR | NR | NR | NR | NR | NR | NR |
| **FL Tool**  Stjernqvist (2021) | V | V | NR | D | NR | NR | V | NR | NR | V | NR | NR |
| **FNLAT**  Ashoori (2020) | V | V | NR | V | NR | NR | NR | NR | NR | NR | NR | NR |
| **THAI-NLAT**  Deesamer (2020) | NR | V | NR | NR | NR | NR | V | V | NR | V | V | NR |
| **Preschool-FLAT** Tabacchi (2020) | V | V | NR | NR | NR | NR | NR | NR | NR | NR | NR | I |
| **TFLAC**  Amin (2019) | NR | V | NR | D | NR | NR | NR | NR | NR | NR | NR | NR |
| **CNL-E**  Naigaga (2018) | V | V | NR | NR | NR | NR | NR | NR | NR | NR | NR | NR |
| **FNLIT**  Doustmohamma-dian (2017) | V | V | NR | V | NR | NR | NR | NR | NR | NR | NR | NR |
| **MBL Tool**  Williams (2017) | NR | V | NR | D | NR | NR | NR | NR | NR | NR | NR | NR |
| **CNL Tool**  Guttersrud (2015) | V | V | NR | NR | NR | NR | NR | NR | NR | NR | NR | NR |
| **FLLANK**  Reynolds (2012) | NR | V | NR | V | NR | NR | NR | V | NR | NR | V | V |
| M-FNLIT, Modified Food and Nutrition Literacy; FNLQ-SC, Food and Nutrition Literacy Questionnaire for Chinese School-age Children; FL, Food Literacy; THAI-NLAT, Thai-Nutritional Literacy Assessment Tool, Preschool-FLAT, Preschool-Food Literacy Assessment Tool; TFLAC, Tool for Food Literacy Assessment in Children; CNL-E, Critical Nutrition Literacy-Evaluation; MBL Tool, Menu Board Literacy Tool; FLLANK, Food Label Literacy for Applied Nutrition Knowledge Questionnaire.  V, Very Good; D, Doubtful; I, Inadequate; NR, Not Reported. Authors changed ‘structural validity’ to ‘structural/construct validity’. | | | | | | | | | | | | |
